# Transcriptome-wide profiling of acute stress induced changes in ribosome occupancy level using external standards

**DOI:** 10.1371/journal.pone.0294308

**Published:** 2023-11-21

**Authors:** Annie W. Shieh, Sandeep K. Bansal, Zhen Zuo, Sidney H. Wang

**Affiliations:** 1 Center for Human Genetics, The Brown foundation Institute of Molecular Medicine, The University of Texas Health Science Center at Houston, Houston, TX, United States of America; 2 Baylor College of Medicine, Houston, TX, United States of America; Carnegie Mellon University, UNITED STATES

## Abstract

Acute cellular stress is known to induce a global reduction in mRNA translation through suppression of cap dependent translation. Selective translation in response to acute stress has been shown to play important roles in regulating the stress response. However, accurately profiling translational changes transcriptome-wide in response to acute cellular stress has been challenging. Commonly used data normalization methods operate on the assumption that any systematic shifts are experimental artifacts. Consequently, if applied to profiling acute cellular stress-induced mRNA translation changes, these methods are expected to produce biased estimates. To address this issue, we designed, produced, and evaluated a panel of 16 oligomers to serve as external standards for ribosome profiling studies. Using Sodium Arsenite treatment-induced oxidative stress in lymphoblastoid cell lines as a model system, we applied spike-in oligomers as external standards. We found our spike-in oligomers to display a strong linear correlation between the observed and the expected quantification, with small ratio compression at the lower concentration range. Using the expected fold changes constructed from spike-in controls, we found in our dataset that TMM normalization, a popular global scaling normalization approach, produced 87.5% false positives at a significant cutoff that is expected to produce only 10% false positive discoveries. In addition, TMM normalization produced a systematic shift of fold change by 3.25 fold. These results highlight the consequences of applying global scaling approaches to conditions that clearly violate their key assumptions. In contrast, we found RUVg normalization using spike-in oligomers as control genes recapitulated the expected stress induced global reduction of translation and resulted in little, if any, systematic shifts in the expected fold change. Our results clearly demonstrated the utility of our spike-in oligomers, both for constructing expected results as controls and for data normalization.

## Introduction

Advances in high throughput gene expression profiling technologies, such as microarrays and next generation sequencing technologies, have revealed many technical challenges in making seemingly straightforward comparisons. Oftentimes, despite great care in implementing experimental procedures, systematic shifts in quantification range are observed between samples, and sometimes even between samples that are otherwise considered identical. In response to these technical challenges, many procedures have been developed to identify and to remove these unwanted variations [1–9]. Approaches such as standardizing read count by library size and global scaling to homogenize quantitative range across samples are among the most commonly used strategies for increasing statistical power to detect differential expression and to improve accuracy in effect size estimates [2, 5]. These powerful approaches, however, make certain assumptions that need to be met in order to deliver the intended results [10]. For example, a global scaling normalization approach, such as TMM, assumes that any systematic shift in quantitative range is an experimental artifact, and scales the data accordingly to mitigate the systematic bias. Such assumption is often valid, as a clear global shift would indicate an unlikely scenario that most, if not all, genes are changing expression level in the same direction relative to the control samples/conditions. In some scenarios, however, a systematic shift in gene expression is a part of the biological process of interest. For example, a key step of the Unfolded Protein Response (also known as Integrated Stress Response) is the phosphorylation of a translation initiation factor eIF2-alpha, which in turn suppresses cap-dependent translation in order to reduce protein synthesis load in the ER [11–15]. In such a scenario, a global reduction in mRNA translation is expected. Applying global scaling normalization on a dataset profiling stress induced changes in mRNA translation level will therefore erroneously normalize away many of the stress induced changes by severely distorting the effect size of most, if not all, quantitated genes.

In a scenario where a global reduction in gene expression is expected, a normalization approach alternative to global scaling is needed to accurately quantitate gene expression. External standards designed for quality control to facilitate cross platform comparison, such as ERCC RNA spike-in control mixes [16, 17], could be used in such experiments to provide reference points for normalization. In theory, a straightforward regression based standard curve approach could connect estimates of gene expression level to RNA concentration (i.e. absolute quantification), however, this approach has been shown to be ineffective for normalization efforts to increase power in differential expression tests: Using libraries constructed with ERCC spike-in pool, Jiang et al. found results from a standard-curve-based normalization to produce significant deviations in the observed fold change from the expected, despite high overall Pearson correlation for spike-in quantification between libraries [17]. The observed deviations exceeded two fold for spike-in controls in the lower abundance range, likely arising simply from sampling variation [17]. In other words, despite achieving overall accuracy, a standard curve approach is not precise enough for detecting biologically relevant fold changes, especially at the extreme ends of the quantitative range.

To provide a feasible alternative, Risso et al. contemplated between different approaches of using spike-in controls for data normalization and demonstrated potential issues with spike-in based global scaling approaches and a spike-in based local regression approach [1, 2, 18]. Instead, Risso et al. adapted a factor analysis approach for control-gene-based normalization (RUVg) by treating quantifications of spike-in oligomers as control genes. RUVg estimates unwanted effects from control genes, agnostically to the effect of origin, and uses these estimates as covariates in a linear model for differential expression tests and for fold change estimates. Using quantification of ERCC spike-in oligomers as control genes, Risso et al. found RUVg to outperform its contemporaries [1].

While ERCC spike-in oligos are available for RNA-Seq studies, they are not easily adaptable to ribosome profiling experiments, an approach enabling transcriptome-wide profiling of mRNA translation [19]. Consequently, transcriptome-wide profiling of translation level changes in response to external stimuli, especially for those that have the potential to induce global shift in quantification, has been challenging [20]. Earlier stress response ribo-seq studies therefore opted for either not normalizing the data or normalizing the data with available methods that were not necessarily suited for the purpose [21, 22]. On the other hand, Andreev et al. used a single oligomer spike-in as external control for data standardization in their study on the impact of Sodium Arsenite treatment (oxidative stress) on mRNA translation in HEK293T cells [23]. Their analysis, however, was limited by a rather small sample size and by their design of a single spike-in oligomer control, which does not enable more rigorous assessments on normalization impact. Work from Iwasaki et al. and subsequently Liu et al used mitochondrial ribosome footprints as controls for normalization and achieved apparent success [24, 25]. However, as was articulated by the authors, the underlying assumptions supporting the use of mitochondrial footprints as controls for normalization may or may not be met in different experimental conditions. Such an approach is therefore unlikely to be generalizable. Even in studies where the results appear to meet expectations, the caveat of observing false results introduced by potential systematic shifts in mitochondrial ribosome footprints could undermine the conclusion.

To meet this apparent need, a few recent studies have explored the utility of different spike-in formulations as external standards for data normalization [26, 27]. Here we report our efforts on this front. We developed and further characterized a panel of 16 spike-in control oligomers designed for ribosome profiling experiments. To characterize these spike-in oligomers and demonstrate their utility in ribo-seq data normalization, we conducted a study to profile transcriptome-wide changes in ribosome occupancy level induced by Sodium Arsenite treatment. Our spike-in formulation and study design provides the expected fold changes between samples, which enables rigorous evaluation of normalization impact on effect size and false positive rate.

## Results

### A set of short spike-in control oligos designed for human ribo-seq experiments

Our spike-in oligomer design follows Lutzmayor et al. oligomer design for small RNA-Seq [28]. The key features are 1. A core sequence at position 5–24 nt that mimics the base composition of the first 20 nts of human miRNA. 2. Four-nucleotide-random-sequences flanking each end. 3. A free energy profile mimicking endogenous miRNA. 4. End modifications to mimic ribosome footprints (S1A Fig in S1 File). We designed the spike-in oligomers based on endogenous miRNA minimum free energy profile, because we frequently observed “ribosome protected fragments” pileup at miRNA loci. While it remained unclear what proportion of these miRNA fragments were true ribosome footprints and what proportion were resulted from miRNA RISC complex co-purified with monosomes during size exclusion column purification, these miRNA fragments clearly made their way into the ribo-seq data. We therefore reason that artificial oligomers mimicking miRNA folding and base composition will make good candidates as spike-in controls for ribo-seq studies.

**Fig 1:**
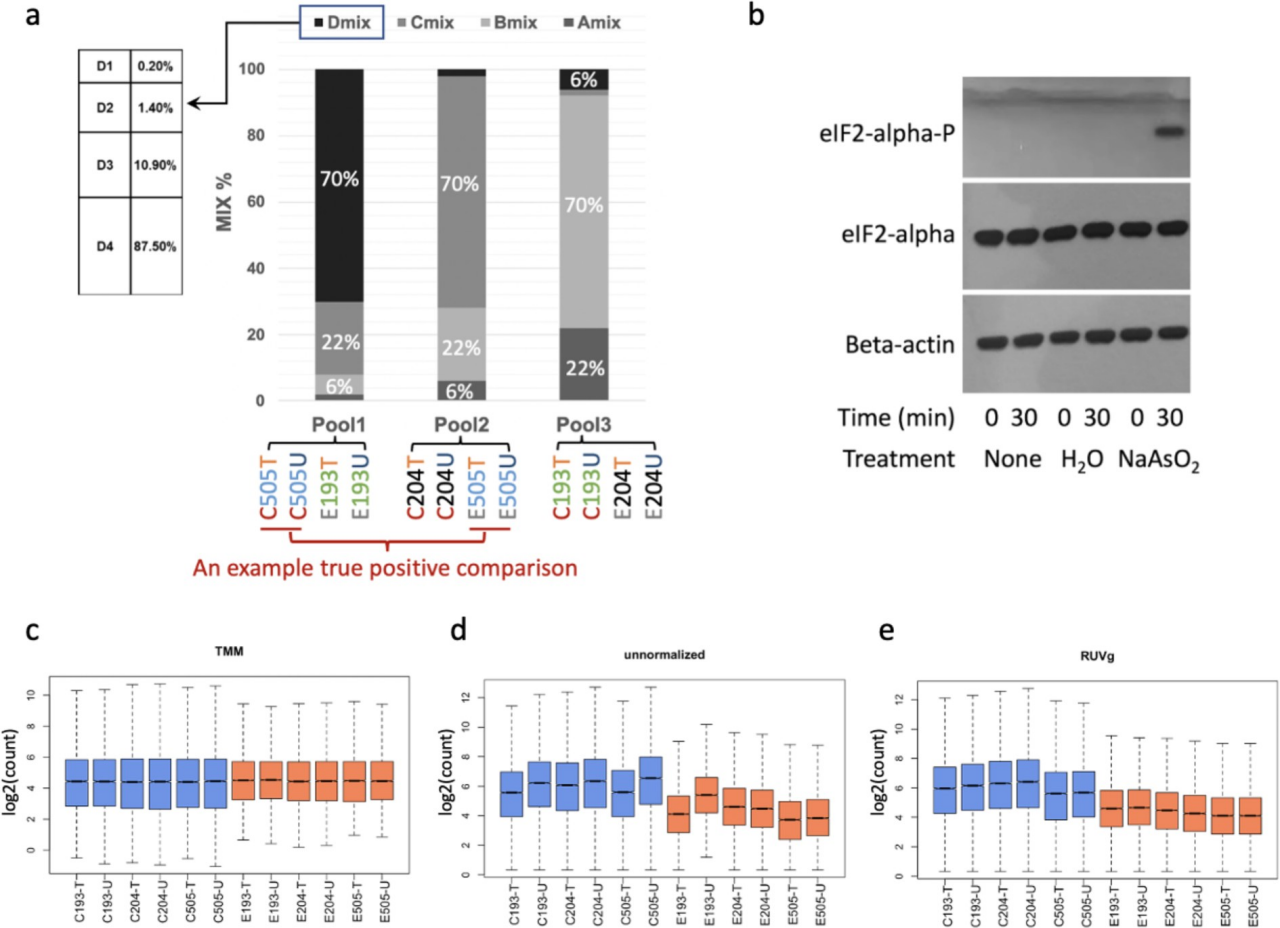
The design and application of spike-in oligomers for stress response ribo-seq studies. (a) Spike-in oligomer pooling design: A stacked barplot showing proportion of spike-in mixes used in each spike-in pool. Each mix is composed of 4 spike-in oligomers (e.g. D1, D2, D3, D4) mixed in the same 8 fold increment from oligomer 1 to oligomer 4 (proportion in percentages presented in the table to the left for mix D). Below the stacked barplot, samples receiving each spike-in pool are labeled with color code distinguishing different effects of interests to highlight the study design. Sodium Arsenite treatment (Control vs. Experiment). Donor (18505 vs. 19204 vs. 19193). Library preparation batch (T vs. U). For true negative and true positive control comparisons we compared quantification of the same oligomer between treated (Experiment) and untreated (Control) cell lines that either received the same oligomer pool (true negative; black brackets) or the cell lines were derived from the same individual but received different oligomer pools (true positive; the red bracket marked one such example), respectively. (b) Western blots for GM18505 indicate that our treatment conditions induced integrated stress response. Primary antibodies used were labeled to the left of the blots. Treatment types and durations were labeled below the blots. Treatment type "None” indicated baseline conditions, while treatment type "H2O” indicated the control condition used in the ribo-seq study. Note the band of phosphorylated eIF2α, a stress marker, only visible in Sodium Arsenite treated samples in contrast to the loading controls. (c, d, e) Boxplots summarizing impact of different normalization strategies on overall distribution of ribosome occupancy level (log2 counts) across all genes analyzed. Boxes are color coded to distinguish control samples (blue) from Sodium Arsenite treated samples (orange). The maximum and minimum values for the boxplot (i.e. the whiskers) are defined by the genes with quantification levels closest to (but without exceeding) 1.5 times of the interquartile range extending from the box.

Using this design, we generated 993 core sequences each in combination with 65,536 different permutations of flanking sequences. We selected 16 oligo sequences, one from each category of core sequence, based on their free energy profile similarity to the endogenous miRNA (S1B Fig in S1 File); 16 different spike-in oligomers were purchased from the manufacturer in two batches of 8 (S1 Table in S2 File). These synthetic oligomers were then mixed to create spike-in pools, following the modified Latin square design by Pine et al. [29], to create pools of spike-in oligos that cover a relative quantitative range of 1~17,920 (Fig 1A). We applied these spike-in pools for ribo-seq experiments by adding the oligo-mix to the RNA sample prepared from the elute of size exclusion column purification of macromolecular complexes (intended to isolate individual ribosomes loaded with footprinting RNA fragments), which we termed digested macromolecular RNA. Because the majority of the digested macromolecular RNA are not ribosome footprints, through trial and error, we empirically determined the ratio between the spike-in oligo pool and the digested macromolecular RNA that will result in a desirable amount of spike-in oligomers sequenced in the final ribo-seq library. We found that, in our system, a 1:20,000 weight ratio between the spike-in pool and the digested macromolecular RNA results in an average of 2.30% final spike-in count/ total footprint count in the sequencing data.

### A stress response ribo-seq study for evaluating spike-in utility

We aim to evaluate the utility of spike-in oligomers in a well-established cell line model system in a scenario where a global shift in translation level is expected. As such, we collected ribo-seq data with spike-in from three unrelated HapMap LCL cell lines (GM18505, GM19193, GM19204) with and without 30 minutes of Sodium Arsenite induced Oxidative stress. A 30 minute treatment with Sodium Arsenite at a final concentration of 134 μM is sufficient to induce phosphorylation of eIF2α in LCL (Fig 1B and S2 Fig in S1 File). Phosphorylation of eIF2α is a hallmark of integrated stress response and is expected to result in a global reduction of cap-dependent mRNA translation [11, 12]. From each sample we created two replicates of sequencing libraries, one of which was treated with an additional CRISPR depletion step aimed to further reduce the level of rRNA contamination in ribo-seq libraries.

We generated a total of 873 million sequencing reads, which, after filtering out the rRNA, tRNA, and snoRNA reads, resulted in an average of ~2 million ribosome footprint reads uniquely mapped to the GRCh38 human genome and ~32K reads mapped to the spike-in oligo sequences (S2 Table in S2 File). Consistent with prior publications, we found more than 80% of the ribo-seq reads are derived from either ribosomal RNA, transfer RNA, or small nucleolar RNA (S3 Table in S2 File). On the other hand, in comparison to our past experience with ligation based ribo-seq protocol, we found the current set of libraries, generated using a ligation free protocol, to have a higher proportion of sorter-than-25-nt ribo-seq reads, which resulted in an overall lower proportion of uniquely mapped reads (S2, S3 Tables in S2 File). Nonetheless, the ribo-seq reads, when viewed in aggregate, show a clear subcodon periodicity pattern in the coding region at the expected positions (S3 Fig in S1 File). A clear pattern of subcodon periodicity reflects the mechanism of ribosomes decoding messages and indicates the quality of our footprinting data. Using principal component analysis (PCA), we found the stress treatment (separated along the first PC, 31.9% variance) and the cell line identify (separated along the second and the third PC, 13.5% and 11.6% variance, respectively) contributed to the majority of variation in our ribo-seq data (S4 Fig in S1 File). We found no significant differences in the proportion of rRNA reads between libraries prepared with and without the CRISPR depletion step (P = 0.82, Wilcoxon rank sum). Libraries with and without CRISPR treatment were then treated as technical replicates in this study to evaluate the unwanted effects introduced from separate rounds of library preparations (i.e. library batch).

### Quantitative properties of spike-in oligo pools

We next evaluate our spike-in oligo pools for their quantitative range and their correlations with expected concentrations. To focus our analysis on quantitated genes, we required, for each GENCODE annotated gene, at least one sequencing read mapped to the gene in each sample of our dataset. With this criteria our sequencing coverage enabled analysis of 12,357 genes. We found that the panel of 16 spike-in oligomers span similar quantitative ranges across samples, which on average covers 97% of the quantitated genes (S5A Fig in S1 File). Without further normalization, we readily observed the expected global reduction in ribosome footprint counts across quantitated genes in the Sodium Arsenite treated samples (S5A Fig in S1 File and Fig 1D). Overall we observe strong positive correlations between spike-in sequencing counts and their corresponding nominal concentrations (median Spearman’s rho at 0.87, ranging between 0.81 to 0.92; S5B Fig in S1 File). Importantly, we found no significant effect from treatment conditions (P = 0.86, ANOVA) nor from technical replication batches (i.e. library batches, P = 0.19, ANOVA) on the correlation between the observed and the expected quantification levels for spike-in oligo pools. On the other hand, we noticed that the oligos ordered from two separate manufacturing batches show clear batch effects (S5C Fig in S1 File; P = 1.43e-4, ANOVA); indicating the importance of considering the batch effect from manufacturing, especially if the oligomers were intended for use in a standard curve.

### Quantitative properties of individual spike-in oligo

**Fig 2:**
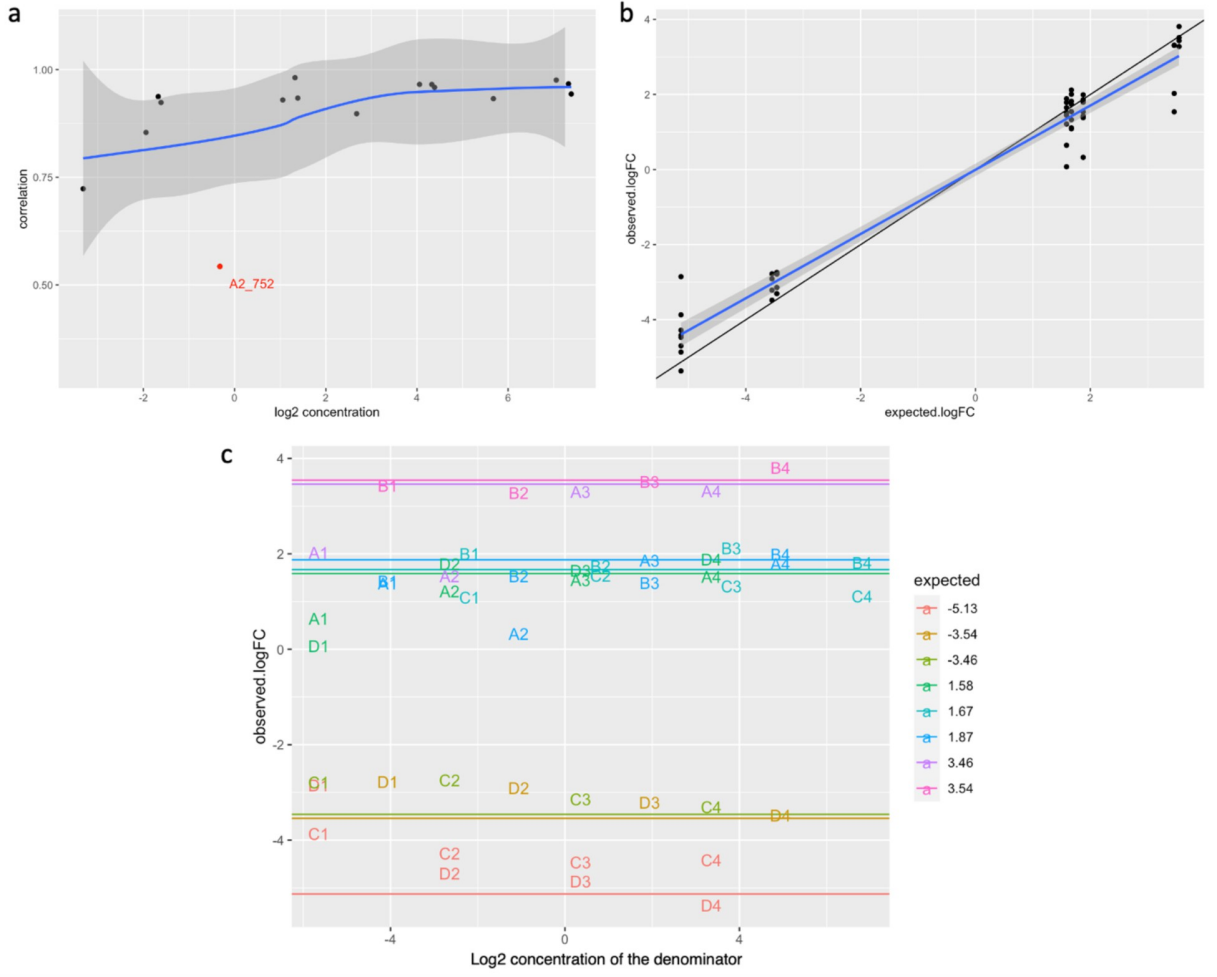
Quantitative properties of spike-in oligomers. (a) Correlations (Pearson) between the observed quantification and expected for each spike-in oligomer is plotted against the acrosspool-average-expected concentration of the spike-in oligomer. Note the downward trend towards the lower concentration range. Blue loess trend line and its corresponding 95% confidence interval (shaded area) indicated oligomer A2 (highlighted in red) as an outlier. (b) Observed log2 fold change for spike-in constructed true positives plotted against the expected. Each data point represents the fold difference of the same oligomer between 2 different spike-in pools. The blue trendline and shaded area represents the linear regression coefficients and corresponding 95% confidence intervals calculated using the expected log fold change as predictor. (c) Observed log2 fold change for spike -in constructed true positives plotted against the expected concentrations of the quantifications used as the denominator for the observed log2 fold change calculation. The horizontal lines mark the expected log2 fold change. Each data point is represented by corresponding oligomer identification abbreviations and color coded in the same way as the expected fold change lines. Note the ratio compression at the lower concentration range.

To evaluate the quantitative properties of each individual oligomer across samples, we first evaluate the monotonicity of oligomer quantification (i.e. whether we observe the same ranking between the expected, based on concentration, and the observed quantification). Using Spearman’s Rho, we found all spike-in oligos used in this study were monotonic. We next evaluated the Pearson correlation between the expected, based on concentration, and the observed quantification level. Overall We found strong correlations between the expected and the observed (median 0.94, interquartile range 0.92~0.97). On the other hand, for spike-in oligos used in the lower concentration range, we observed a decrease in correlation between the observed and the expected (Fig 2A). This decrease in correlation is likely reflecting the increased coefficient of variation (i.e. increased variance relative to the mean) commonly observed in RNA-seq data at the low count region (i.e. an over-dispersed Poisson).

Of note, we found oligo A2_752 to be an outlier of the observed trend (Fig 2A). A Pearson correlation between the observed quantification and the expected based on input concentration for A2_752 is 0.54, which fell far below the 95% confidence interval of a loess fit adjusting for the concentration range effect. Upon further exploration of the properties of oligomers, we found A2_752 to have the highest minimum free energy of 0 among the 16 spike-in oligomers. On the other hand, we found no significant association between oligomer minimum free energy and the Pearson correlation between the observed and the expected oligomer quantification (P = 0.60, S6 Fig in S1 File).

Next, for each spike-in oligomer we compare the observed between-sample fold change to the expected fold change, which is calculated based on our modified Latin square pooling design (Fig 1A). Overall, we found a strong positive correlation with a small ratio compression effect in the observed fold change relative to the expected (Fig 2B). When using the expected fold change of spike-in oligomers in a linear model to predict the observed fold change, we found a regression slope of 0.87 +/- 0.023, which is close to but significantly different from the expected regression slope of 1 (P = 2.28e-6). This ratio compression effect appears to be stronger in the lower concentration range (Fig 2C). In fact, by stratifying data into three groups of the concentration range covered in our study, we found a progressive increase in ratio compression in the lower concentration group (S7 Fig in S1 File), which points to the possibility of an additive process such as consistent pipetting bias during serial dilution as the cause of the observed ratio compression. On the other hand, the overall strong correlation observed without normalization provides strong support for the high technical consistency of our experimental operations and our choice of loading reference.

### Spike-in based data normalization preserved the expected global shift in quantification while global scaling normalization produced high level of false positives

It has previously been shown that a standard-curve-based-normalization approach using control oligos is technically challenging [17]. Consistently, evaluation of our spike-in oligos has shown both manufacturing batch effect and ratio compression effect, each as a practical example of the technical challenge of a standard-curve-based approach for absolute quantification. Alternatively, Risso et al. have developed a factor-analysis-based approach—RUV, which uses “control genes” for normalizing RNA-seq data [1]. Here using our spike-in oligomers as control genes we applied RUVg for an external-standard-based normalization of our ribo-seq stress response dataset and compared the results with those that we generated from applying a popular global-scaling-normalization approach, TMM [2]. Note that as a result of adapting the RUVg approach, despite using external standards, our approach doesn’t provide absolute quantification, instead it provides between condition fold changes (i.e., relative quantification), which is estimated from sequencing counts after accounting for the unwanted factors.

The first, and often challenging, step of applying RUVg for data normalization is to determine the number of unwanted variables (k) to remove. As a factor analysis method, RUVg approaches the data normalization issue by identifying unknown factors that contribute to the inter-sample distribution differences in quantification level. These nuisance variables, such as variation in sequencing depth, technical differences between batches or between sequencing centers, that contribute to unwanted variation are termed unwanted variables (k). While RUVg provides a formal procedure for estimating these unwanted variables, it is not always clear how many of these unwanted variables should be removed. Removing too many k could end up inadvertently removing biological variations and, in some designs, overfit the data.

Our external spike-in provides a principled approach to determine k. We evaluated the impact of removing unwanted variables on the correlation between the observed spike-in quantification and the expected. By incrementally removing unwanted variables, we found that the increased correlation between the observed and the expected spike-in quantification started to plateau at k = 3. We therefore decided on removing the top 3 unwanted factors, which increased the correlation between the observed spike-in quantification and the expected from R^2^ = 0.73 to 0.83. This choice of k is robust against the exact composition of spike-in oligomers used for normalization (S8 Fig in S1 File). Using spike-in based RUVg normalization, we found the resulting normalized data to preserve the expected global reduction in ribosome occupancy level (Fig 1E). In contrast, when applying TMM normalization to the same dataset, the expected global shift is lost (Fig 1C).

The loss of an expected global shift will clearly result in false negatives. On the other hand, we suspect the same underlying shift in expression level created by TMM method could also introduce false positives. To evaluate the extent of false positive discoveries in such a scenario, we constructed true negative comparisons using quantifications for the same oligos between samples receiving the same pool of spike-in oligomer mixtures (black brackets in Fig 1A) and performed tests for differential expression between control and treated (e.g. comparing D1 quantifications between C505 and E193 samples; Fig 1A). We compared the number of discoveries between unnormalized data, TMM normalized data, and RUVg normalized data. At a P value cutoff of 0.1, which is expected to result in 10% false positives by chance, we found ~20% false positives from tests using either unnormalized data or RUVg normalized data. Conversely, at the same 10% cutoff, using TMM normalization we found 42 false positives out of a total of 48 tests (87.5%). The extremely high proportion of false positives identified using TMM normalized data is in clear contrast to the proportion of false positives identified either using unnormalized data or RUVg normalized data (S9 Fig in S1 File).

### Global scaling normalization introduced false positives in stress response dataset by distorting fold change

**Fig 3:**
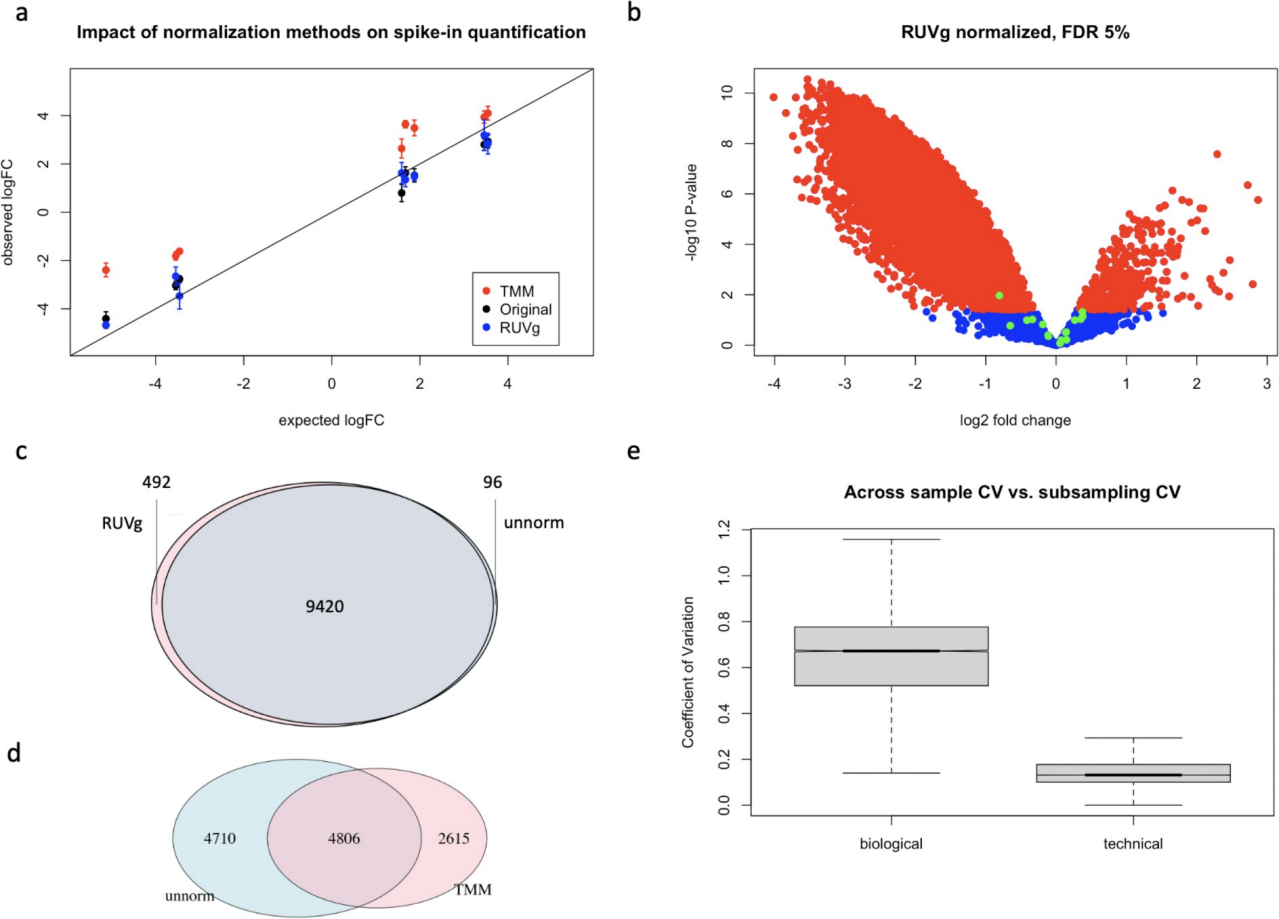
Impact of TMM normalization and spike-in based RUVg normalization on expression quantification. (a) Observed log2 fold change for spike-in constructed true positives plotted against the expected; comparing between results from different normalization approaches. Data points represent mean plus/minus standard errors calculated from each group of true positives that share the same expected log2 fold change. Black data points (i.e. "Original”) are results from log2 transformed counts without further normalization. Black line indicates the ideal correlation (i.e. an intercept of 0 and a slope of 1). (b) A volcano plot showing the relationship between fold change (treatment versus control) and p value from differential expression tests for RUVg normalized data. Data points are color-coded in green for spike-in oligomers, in red for endogenous genes that are significantly differentially expressed at 5% FDR, and in blue for endogenous genes that are not differentially expressed. (c, d) Venn diagrams summarizing the number of shared and distinct differentially expressed genes found with and without normalization. Unnormalized (unnorm) in blue. Normalized (either TMM or RUVg) in pink. Numbers labeling each area indicate the number of genes belonging to each group, with the size of the area drawn in proportion to the size of the group. (e) Spike-in based RUVg normalization results are robust against the exact set of spike-in oligomers used as control genes. Boxplots comparing between the across sample biological CV to the across subsampling technical CV for endogenous genes. The technical CV here is calculated based on the normalized quantification level of endogenous genes across 10 iterations of spike-in oligomer subsampling for control gene selection.

To identify the underlying cause of false positive and false negative results introduced by TMM normalization of our stress response dataset, we used the input concentration of spike-in oligomers to construct true positive comparisons with expected fold changes. More specifically, we used quantifications of the same spike-in oligomer from cell lines derived from the same individual but received different pools of spike-in oligomer mixtures (see red brackets in Fig 1A for an example) and performed tests for differential expression between control and treated (e.g. comparing D1 quantifications between C505 and E505 samples). Using the expected fold change calculated from known oligomer concentrations for the true positives, we found TMM normalization to clearly distort the fold change (Fig 3A and S10 Fig in S1 File). When modeling the observed log2 fold change in TMM normalized spike-in quantification using the expected log2 fold change as the predictor, we found TMM normalization to shift the log2 fold change of spike-in quantification from the expected by 1.612 +/- 0.119 (i.e. a systematic upward shift) while maintaining a regression coefficient of 0.824 +/- 0.038 and an r-squared of 0.910 (P < 2e-16; Fig 3A). In contrast, only -0.082 +/- 0.105 log2 deviations from the expected fold change were observed for unnormalized data, with a comparable regression coefficient of 0.832 +/- 0.033 and an r-squared of 0.930 (Fig 3A).

The distortion in fold change estimates observed in TMM normalized data explains the high false positive rates observed in the aforementioned true negative control comparisons and indicates the possibility of introducing false negatives by the global scaling normalization approach shifting true differences towards zero. On the other hand, RUVg normalization appears to have nudged the observed fold change towards the expected (Fig 3A), which resulted in a slight increase in the regression coefficient, from 0.832 (+/- 0.033) of the unnormalized to 0.885 (+/- 0.039) and the estimated intercept changed from -0.082 (+/- 0.105) of the unnormalized to -0.044 (+/- 0.123), both parameter values inching closer to the expected (i.e. a slope of one and an intercept of zero).

### Spike-in based RUVg normalization recovered 99% of the discoveries from unnormalized data and identified an additional 5% of differentially translated genes

After evaluating the impact of normalization on spike-in oligomer quantification, we next evaluated the impact of different normalization procedures on the 12,357 endogenous genes that were sufficiently quantitated. We compared test results for identifying differentially translated genes between control and treated samples across datasets normalized using either a spike-in based RUVg normalization or TMM normalization. In these comparisons, we evaluate two main aspects of data normalization impact, namely, improvement in detection power and introduction of biases in effect estimates. Raw data collected without biased manipulations reflects true effects buried in both technical and biological noises. In other words, assuming no biased manipulations involved, effects estimated from unnormalized data are accurate but imprecise. Data normalization, when applied appropriately, should increase power without introducing bias. Using spike-in based RUVg normalized data, at 5% FDR, we identified 9,912 stress-induced differentially translated genes. Consistent with the observed global reduction in translation activities, 96% of stress induced changes were down regulations (Fig 3B). When compared with test results from unnormalized data, RUVg normalization replicated 99% of the discoveries (i.e. 9,420 out of 9,516) and identified an additional 492 genes (~5%) (Fig 3C); a moderate increase in power. In contrast, using TMM normalization, at 5% FDR, we identified 7,421 stress-induced differentially translated genes. Of these differentially translated genes only 48% were down regulations (S11B Fig in S1 File). When compared with test results from unnormalized data (Fig 3D and S11 Fig in S1 File), TMM normalization only replicated 51% of the discoveries (i.e. 4,806 out of 9,516). On the other hand, 2,615 of the differentially translated genes found in TMM normalized data (i.e. 35% of TMM discoveries) were not found in test results derived from the unnormalized data (Fig 3D). The rather limited overlap between differentially translated genes identified from unnormalized data and the ones identified from TMM normalized data indicates potential biases introduced from TMM normalization.

### Global scaling normalization of stress response datasets leads to high rates of false discoveries among endogenous genes

Following our observation with spike-in oligomers, we consider the possibility that TMM normalization introduces biases among endogenous genes through the same upward shift of quantifications in treated samples (Fig 3A). Indeed, for genes that were found differentially translated in unnormalized data but not in TMM normalized data (i.e. potential false negatives), we found TMM normalization to shift the log2 fold change towards zero, which resulted in a shift of log2 fold change by 1.733 +/- 0.001 (treated samples relative to control sample; S12A Fig in S1 File). This upward shift is comparable in magnitude to the upward shift observed in spike-in oligomers (1.612 +/- 0.143) (S10 Fig in S1 File). This systematic shift is not limited to those potential-false-negative genes, rather the systematic shift can be clearly visualized when we plot the log2 fold change of TMM normalized data against the unnormalized data for each individual gene across all quantitated genes (S12B Fig in S1 File).

In addition to introducing false negatives, a systematic upward shift could also produce false positives (i.e. by shifting true negatives away from zero). In such a scenario, these differences in discoveries between TMM normalized data and unnormalized data were resulted from biased effect estimates instead of a difference in statistical power. In other words, we do not expect relaxing significance cutoffs to recover these mismatches. Consistent with our expectations, for differentially translated genes found in TMM normalized data (at 5% FDR) but not in unnormalized data, we found limited recovery from relaxing significance cutoffs for unnormalized data (S12C Fig in S1 File). For example, when relaxing significance cutoff for testing differential expression in unnormalized data to 20% FDR, we recovered only 25.6% of the TMM-normalization-specific findings. Importantly, amongst the recovered genes, 69.4% had an opposite direction of effect between datasets indicating that for these genes TMM normalization distorted the original effect to an extent that flipped the sign. In contrast, for RUVg normalization, we found a much higher recovery rate (S12D Fig in S1 File). Using 20% FDR for the unnormalized data, we recovered 86% of the normalization specific findings. All of the recovered effects were found in the same direction between the unnormalized data and RUVg normalized data. Taken together, these results highlight the issues in using a global scaling approach, such as TMM, to normalize ribo-seq datasets in scenarios of an expected global shift in mRNA translation, such as acute cellular stress response.

### Spike-in-based RUVg normalization results are consistent regardless of the exact combinations of spike-in oligomers used

To evaluate the robustness of spike-in-based RUVg normalization, we took a subsampling approach. For each iteration we randomly subsampled half of our spike-in quantification data as control genes. We compared RUVg normalization results across ten iterations of subsampling. We first consider the variation in normalized quantifications of spike-in oligomers across iterations of subsampling. For the spike-in comparisons designed to have expected fold changes (i.e. true positives) we found the observed fold changes to have a tight range of values across subsampling iterations (S13A, S13B Fig in S1 File). The deviation of the observed log2 fold change from the expected is centered at zero with the percentage of deviation consistently at around 30% across iterations (33.8% +/- 0.3% of the expected). Similarly when using the expected fold change as the predictor in a linear model for the observed, we found rather consistent regression coefficients across subsampling interactions and the intercept to center at zero (regression coefficients: 0.888 +/- 0.002, intercept:-0.016 +/- 0.027) (S13C, S13D Fig in S1 File). For the true negatives, we found the distribution of fold change to consistently center near the expected zero across iterations (S14A Fig in S1 File) and the proportion of false positive discoveries to consistently fell at ~10% above the expected (S14B Fig in S1 File), which is similar to the results from normalizing using the full set of spike-in oligomers (S9C Fig in S1 File). Qualitatively similar, albeit noisier, results were observed when analyzing only spike-in quantification data that were not included as control genes for normalization (fold change: -0.021 +/- 0.045, proportion false positives: S14C Fig in S1 File).

We next consider the variation in normalized quantifications of endogenous genes across iterations of subsampling. We found an average CV of 14.7% across subsampling iterations (interquartile range: 10% to 17.7%) and the CV decreases steadily as a function of quantification levels (i.e. log2 counts; S15A Fig in S1 File). These properties are similar to the technical variations observed in published RNA-Seq data [30]. When comparing the subsampling CV to the across-sample-biological CV (i.e. variations mainly originated from the between treatment group differences) we found the across-sample-biological CV to be more than 4 times higher than the subsampling CV (average at 64.7% vs. 14.7%; P < 2.2e-16; Fig 3E). When limiting the CV comparison to genes with low quantification levels (here we set the quantification cutoff at an average count of less than 32), from which we often see strong overdispersion, we found similar large differences between the biological CV and the subsampling CV (58.5% vs. 15.4%; P < 2.2e-16; S15B Fig in S1 File). The large difference between biological CV and subsampling CV indicated that subsampling variations likely had limited contribution to false discoveries, which is in agreement with the stable false positive rates observed across iterations of subsampling (S14 Fig in S1 File). Taken together these results indicated that the spike-in based normalization approach developed here is robust against the exact combination of spike-in oligomers used for the purpose of identifying stress induced differences in mRNA translation.

## Discussion

We developed a panel of 16 spike-in oligomers and a corresponding pooling scheme for applications in ribosome profiling studies to identify acute cellular stress induced changes in mRNA translation across the transcriptome. To evaluate the utility of this set of spike-in oligos, we performed ribosome profiling experiments to identify Sodium Arsenite induced changes in translation level from three LCLs in two conditions, each with two library preparation replicates. Note that the changes in ribosome occupancy level observed here is used as a proxy for the changes in the level of translation, which reflects the combined effect of both transcriptional and translational regulation of gene expression.

Evaluation of spike-in oligomers overall found strong positive correlations between the observed and the expected. At the same time, we identified one oligomer, A2, as potentially problematic. Because of the fact that our design requires a consistent length and has a rather homogeneous base composition across oligomers, we were unable to identify length bias or sequence features associated with unwanted effects. On the other hand, our spike-in oligomers were designed to span a spectrum of minimum free energy (i.e. a numeric proxy for oligomer folding structure) that resembles endogenous miRNAs, which allows us to evaluate the correlations between minimum free energy and the quantitative properties of the spike-in oligomers. We found no significant association between oligomer minimum free energy and the correlation between the observed and the expected oligomer quantification. A lack of correlation observed between spike-in performance and minimum free energy is reassuring, in that the quantification level is in general not biased by the minimum free energy of RNA fragments. On the other hand, we found the outlier A2 oligomer to have the highest minimum free energy of zero among the 16 spike-in oligomers. Unfortunately, because A2 is the only spike-in oligomer with a minimum free energy of zero, we were unable to conclude if a minimum free energy of zero led to the lower correlation between the observed and the expected.

Our analyses on the quantitative property of spike-in oligomers revealed two major unwanted effects, a manufacturing batch effect and a ratio compression effect.

Based on the observed pattern of manufacturing batch effect, we postulate that the between batch variations in either the quantification process from the manufacturer or variations in preparing the stock solution when we first received the oligomers (e.g. different sets of pipetman used or pipetman calibration shifted over time) were the likely culprit. The progressive nature of ratio compression led us to postulate that a consistent pipetting error could have resulted in the observed compression, which is more pronounced at the lower concentration range. These unwanted technical effects will clearly have a negative impact on a study relying on a standard-curved-based absolute quantification approach. On the other hand, their potential impact on results from a factor-analysis-based relative quantification approach is expected to be limited: unwanted effects that are not shared by endogenous genes, especially the ones that are properly randomized across the effect of interest, such as the oligomer manufacturing batch effect and ratio compression effect observed here, will have limited contribution to the test results when fitted as covariates in the linear model for differential expression.

In contrast to features that are unique to spike-in oligomers, features that are shared between spike-in oligomers and endogenous genes are the critical attributes forming the basis of spike-in-based RUVg normalization. RUVg has previously been used for spike-in-based normalization [1]. Risso et al. have shown that using ERCC spike-in as control genes for RUVg normalization appeared to outperform other popular methods, despite the fact that certain key assumptions of the RUVg normalization method were violated [1]. Following work from Risso et al. we used RUVg to perform spike-in-based data normalization. In the RUVg framework, these spike-in oligomers are treated as control genes and the differences in quantification levels found between samples in these control genes are used to estimate unwanted factors. A key assumption made by this approach is therefore that the spike-in oligomers shared the same impact from the unwanted factors with the endogenous genes. As an example of unwanted effects, we visualized the impact of library preparation batch effects comparing between spike-in oligomers and endogenous genes (S16 Fig in S1 File). Overall, we found a rather similar trend between spike-in oligomers and endogenous genes, with the exception of a bump in the middle of the distribution (S16 in S1 File), which could potentially be attributed to sampling variation.

Applying RUVg normalization to our stress response ribo-seq dataset, using our 16 spike-in oligomers as control genes, preserved the expected global reduction in mRNA translation in response to stress treatment and slightly increased power for detecting differences. Although the power increase was rather limited, RUVg normalization increased accuracy in fold change estimates, as was indicated by the increase in regression coefficient (i.e. approaching the expected value of one) of a linear model fit between the observed and the expected fold changes for control spike-in oligomers. This regression coefficient increase was observed consistently across subsampling from groups of spike-in oligomers. As the first study evaluating the panel of spike-in oligomers, we paid careful attention to avoid introducing biases. Importantly, we did not attempt to increase sequencing coverages only for libraries that appear to have lower coverages (a rather common practice for sequencing studies). This is especially relevant for our study, because most (if not all) of the low coverage libraries are from the treated group; by submitting only libraries from the treated group for additional rounds of sequencing we will introduce bias in the data. Our careful experimental design and operation in conjunction with the large effect from stress treatment likely resulted in the majority of differentially translated genes readily detectable without normalization, which could explain the limited power increase from RUVg normalization. On the other hand, deliberately introducing sequencing coverage differences between samples in future studies will be useful for evaluating the robustness of our spike-in-based RUVg normalization approach against such bias.

A fundamental feature of our study design is the expected fold change of the same oligomer between samples receiving different pools of spike-in oligomers. This expected fold change (or the lack of it) is determined during spike-in pool preparation and this determination is independent of the samples and the downstream experimental conditions. We used the expected fold changes to construct true negatives and true positives, which we used as the yardstick to evaluate our results from quantitating spike-in oligomers. An underlying assumption of this approach is an unbiased (e.g., equal) loading of spike-in oligomer pools between samples. An important decision we made for this study is therefore the loading reference used to determine the amount of spike-in pools to add to each sample. Given that we are working with cultured cells and are testing for differences in cellular response to acute stress, a straightforward choice for loading reference would be the number of cells in each sample. However, our preliminary results indicated high variability between cell counts. Instead, we used the amount of digested macromolecular RNA as our loading reference, which assumes no stress-induced macromolecular RNA turnover within the 30-min treatment time window. Our choice of loading reference is supported both by the literature and by our results from quantitating the between-pool fold change for each spike-in oligomer, which, as mentioned above, is independent of the choice of loading reference. Although, in theory, the amount of loading control used could be determined in absolute scale (i.e. final molar concentration of spike-in oligomers) and the between sample loading variation could then be estimated as unwanted factors by RUVg. In reality, how such an approach would perform requires further investigation with a different study design.

Our spike-in oligomers, when used as control oligos, clearly demonstrated the dramatic consequences of using a global scaling normalization approach to transform ribo-seq data collected from an acute cellular stress response study. When evaluating the expected true negatives constructed based on our spike-in pooling design, we found an extremely high proportion of false discoveries in TMM normalized data. In contrast, in data without normalization or normalized using the spike-in-based factor analysis approach we found the proportion of false discoveries to consistently fall slightly above the expected. When evaluating the expected fold changes of true positives, we found TMM normalization to shift fold changes based on differences in sequencing coverage between libraries. In the current dataset, such shifts ended up creating both false positives and false negatives. We want to emphasize here that the troubling results observed from TMM normalization does not indicate problematic behaviors of the TMM normalization method per se. Instead, it reflects the consequences of violating an important underlying assumption for all global scaling normalization approaches, i.e. any systematic shifts were assumed to be experimental artifacts. In contrast, a factor-analysis-based normalization approach doesn’t stipulate such an assumption. In our analysis, using spike-in oligomer quantification as controls genes, RUVg normalization maintained the expected global reduction of ribosome occupancy level in the treated samples, despite heavily transforming the data (i.e., removing the top three unwanted factors).

In order to keep the linear model parameterization consistent across comparisons, in our differential expression tests, we extracted normalized counts from RUVg as input data. While we designed our study this way to keep a fair comparison between normalization approaches, for studies focusing on identifying genes that are differentially translated, we suggest following RUVg authors’ advice to fit these unwanted factors as covariates. By extracting the normalized counts (i.e. equivalent to regressing out the unwanted factors), one risks potentially removing biologically relevant variations. These considerations are not unlike the ones to contend with when using a typical linear modeling approach for adjusting batch effects. Similarly, when deciding on the number of k to fit as covariates, the degrees of freedom available from the dataset could put some constraints on the available options. The costs and benefits of including additional samples should therefore be carefully considered during study design in order to enable effective factor-analysis-based normalization.

While our study aimed specifically to address the unmet need of external standards for transcriptome-wide profiling of translational regulation in stress response, the exceedingly high proportion of false positives observed as a result of applying TMM normalization to our oxidative stress response ribo-seq dataset and the almost ubiquitous use of global scaling normalization approaches in the field of genomics prompted the following unsettling question: How common have such error occurred without the researchers acknowledging it? It is easy to envision scenarios of subtler global shifts in gene expression for studies comparing between developmental stages or testing for drug treatment effects. Without appropriate use of external standards, these subtle shifts could escape researchers and the subsequent application of global scaling normalization will lead to distortion of effect size, which, as we have shown, results in both false positives and false negatives. On the other hand, while an appropriate application of external standards could safeguard against such pitfalls, further studies to identify effective approaches for determining the amount of spike-in oligomers to use for each sample is needed. Nonetheless, a collective consciousness in reducing experimental biases and a wide adoption of external spike-in oligomers could enable future retrospective meta-analyses to provide insight.

## Materials and methods

### Cell culture and oxidative stress

Three lymphoblastoid cell lines (LCLs) (GM18505, GM19193, and GM19204), each derived from a separate Yoruba people from Nigeria, were purchased from Coriell Institute for Medical Research (NIGMS Human Genetic Cell Repository). The cells were maintained at 37°C with 5% CO_2_ in RPMI media supplemented with 15% FBS, 2 mM L-glutamate, 100 IU/ml penicillin, and 100 μg/ml streptomycin, in accordance with instructions provided by Coriell. Of note, cell cultures were vigilantly maintained at a cell density between 600,000 to 700,000 cells/ml to avoid inadvertent induction of stress response. To induce oxidative stress, cells were treated with 134 μM (i.e. the final concentration in cell culture) Sodium Arsenite (NaAsO_2_; Sigma-Aldrich, Cat # S7400), a heavy metal oxidative stressor, for 30 minutes in otherwise the same cell culture conditions. For the control group, nuclease free water was used in place of the Sodium Arsenite solution. After treatment, cells were pelleted by centrifugation at 100g for 10 min and washed twice with cold PBS (4°C). Cell pellets were flash frozen in liquid nitrogen and stored at -80°C.

### Western blot

Proteins were prepared from flash frozen cell pellets using M-PER Mammalian Protein Extraction Reagent (Thermo Scientific, Cat # 78503) following vendor’s instructions. Protein concentration was estimated using bicinchoninic acid (BCA) assay (Pierce™ BCA Protein Assay Kit, Cat# 23227) and 5 μg of proteins from each sample were used for western blot. All antibodies (e.g. anti eIF2α, anti Phospho-eIF2α) used were purchased from Cell Signaling Technologies (S2 Table in S2 File). XCell II Blot module was used for wet electrophoretic transfer of proteins from SDS-PAGE gel to nitrocellulose membrane (Biorad, Cat # 1620145, 0.45 μm) in Tris-Glycine Electroblotting buffer (National Diagnostic, Cat # EC-880). ProtoBlock Solution (National Diagnostics, Catalog number: CL-252) was used for blocking the nitrocellulose membrane following manufacturer’s instructions. After wet transfer, membranes were incubated with ProtoBlock solution for 1 hour at room temperature before overnight incubation with primary antibodies at 4°C (in blocking solution). After primary antibody incubation, membranes were washed in TBST buffer for three times (5 minutes each) before incubation with HRP conjugated secondary antibody for 2 hours at room temperature (in blocking solution). After secondary antibody incubation, membranes were washed three times in TBST buffer for 5 minutes each. ProtoGlow ECL (National Diagnostics, Cat # CL-300) and HyBlot CL autoradiography films (Thomas Scientific, Cat # NC1601219) were used for signal detection. Quantification of digital images was performed using ImagJ.

### Spike-in oligomers

Spike-in oligomer sequences were designed following Lutzmayer et al. [28]. While we followed the design principle and the programming scripts developed by Lutzmayer et al. a few key aspects were modified to suit our purpose of using these oligomers as external standards in ribosome profiling experiments for human samples. Instead of mimicking the free energy profile and base composition of *Arabidopsis* miRNA, our design mimics human miRNAs. In addition, we extended the oligomer length to 28 nts with flanking random tetramers to mimic ribosome footprint length. We generated/selected 1000 permutations of 20 nts RNA sequences that have a base composition resembling human miRNA (calculated based on a high confidence set of 896 miRNA sequences downloaded from miRBase [31]). Seven of these permutation sequences mapped to the human genome and were therefore removed. For the remaining 993 permutations of RNA sequences, we added random tetramers to each end of the sequence, which resulted in 993 sets of 65,536 sequences. Using RNAfold [32], we determined the minimum free energies of all sequences. Based on the minimum free energy profile, we selected a total of 16 sequences (S1 Table in S2 File), each from a different set, to produce spike-in oligomers for the current study. These 16 spike-in oligomers were purchased in two separate batches (S1 Table in S2 File) from Sigma Aldrich and resuspended in 10 mM Tris (pH 8) to a stock concentration of 100 μM based on the quantifications provided by the vendor. To create the final spike-in pools used in experiments, four spike-in mixes (A, B, C, D) each composed of 4 different oligomers (1,2,3,4) in eight-fold concentration increments were created separately through 2-fold serial dilutions. The four mixes (A, B, C, D) were then combined in a defined ratio of (2%,6%,22%,70%) in 3 permutations to create 3 different spike-in pools each have individual oligomers in different concentrations but together covering the same concentration range (Fig 1A). The working solution for spike-in pools was prepared in a weight concentration of 50 pg/μl, which we found convenient for an application of 50 pg spike-in pool for each 1μg of digested macromolecular RNA before gel isolating the ribosome footprints.

### Ribosome profiling

Ribosome footprint profiling experiments were performed following the ligation free protocol described in Hornstein et al. [33] with a few specific modifications made for the current study. Key steps include, RNase I digestion to generate ribosome protected fragments (100 U per 200 μl of cell lysate), size exclusion spin column (Sephacryl S400, GE: 27-5140-01) isolation of ribosomes, spike-in oligomer control addition, gel isolation of ribosome footprints, sequencing library construction using Clontech SMARTER smRNA SEQ KIT (Fisher Scientific: NC1098027), rRNA depletion using subtraction oligo [34], and finally PCR amplified Indexed libraries were pooled to sequence on an Illumina HiSeq 4000. For incorporating spike-in oligomers, for each sample, 50 pg of spike-in pool was used for each 1μg of digested macromolecular RNA.

### CRISPR/Cas9 targeted depletion of rRNA

CRISPR/Cas9 mediated rRNA removal was performed following Han et al. [35] and Mito et al. [36]. Ten target-specific oligomers for rRNA (see sequences in Han et al. [35]) were ordered from Sigma Aldrich and separately PCR amplified to prepare each oligomer as the template for in vitro transcription (T7-Scribe™ Standard RNA IVT, CELLSCRIPT, Cat# C-AS3107) to produce guide RNAs. CRISPR/Cas9/gRNA solution for the targeted removal of rRNA sequence was prepared and applied to ribo-seq libraries following Mito et al. [36].

### Data processing

#### Sequencing read processing and mapping

Sequence reads were processed and mapped to the human genome following a modified procedure used by Ingolia et al. [34]. Before aligning to the genome, the adapter and polyA sequences as well as 4 nucleotides at the 5’ end were removed from each read using FASTX-Toolkit. Processed reads mapped to a reference FASTA file composed of human rRNA, tRNA and snoRNA sequences were removed. The remaining sequence reads were mapped to the GRCh38 human genome using TopHat2 [37] with splice-junction information from GENCODE GTF (release 37). The mapping procedure allowed a maximum of 2 mismatches and only uniquely mapped reads were retained. Levels of mRNA translation were estimated by counting the number of ribosome profiling reads aligning to each gene based on GENCODE annotation (i.e. the ENSG entries) using featureCounts [38].

#### Data filtering and transformation

To focus our analysis on sufficiently quantitated genes, we only analyzed genes containing at least one sequencing read from each sample, which resulted in a dataset including 12,357 genes (Data S1). For spike-in oligomers, all available quantifications were included in the analyses. Unless otherwise specified, before downstream analyses, count data were log2 transformed after the addition of 0.25 pseudo-count to avoid creating singular values in log scale.

#### Analyses

Statistical analyses were performed in the R statistical computing environment (version 4.0.4). Linear regression analyses were performed using the lm() function. Spearman and Pearson correlations and corresponding tests were performed using cor() and cor.test(). Student’s t-Test were performed using t.test(). Wilcoxon Rank Sum tests were performed using wilcox.test().

### Normalization

TMM normalization was performed using the cpm() function from the edgeR package [39] with effective library size calculated using the calcNormFactors() function. Default parameters were used for the trimmed mean of M value calculation.

RUVg normalization was performed using the RUVSeq package [1]. Factor analysis for identifying unwanted variables was performed by applying the RUVg() function on raw ribo-seq counts using the default settings with the spike-in oligomers as control genes. Note that the factor analysis was done on count data after filtering out genes that do not have at least one count per sample, but without the commonly applied preprocessing of upper quartile normalization, which could remove biologically meaningful coverage differences resulting from the stress treatment. RUVg normalized counts were extracted using the normCounts() function for downstream analyses to visualize the impact of normalization.

### Differential expression tests

Differential expression analyses were performed using the limma package [40]. After processing with various normalization and transformation procedures, functions lmFit() and eBayes() were applied sequentially to perform differential expression tests under a linear modeling framework. When testing for treatment effect for endogenous genes, both library construction batch and donor identity were fitted as covariates in the linear model. More specifically, for each gene, expression level E across sample j is modeled by the treatment effect T as the predictor and the library batch effect B and the cell line effect C as covariates in the following equation:

$$E_{j}=\mu +\beta_{1}T_{j}+\beta_{2}B_{j}+\beta_{3}C_{j}+\varepsilon_{j}$$

Where *μ* is the intercept term and *ε* is the residual. To identify differentially expressed genes, we tested the null hypothesis of *β*_1_ is zero using the empirical Bayes moderated t-statistics from the eBayes() function. False discovery rates were calculated using the Benjamini-Hochberg procedure.

## Supporting information

S1 File Supplemental S1 to S16 Figs. (PDF)

S2 File Supplemental S1-S3 Tables. (PDF)

S1 Data Ribo-seq count table for all genes analyzed. (XLSX)
