# Supplementary material for: Transcriptome-wide profiling of acute stress induced changes in ribosome occupancy level using external standards
Source: PLoS One. 2023 Nov 21;18(11):e0294308. doi: 10.1371/journal.pone.0294308 (PMC10662766; doi:10.1371/journal.pone.0294308)
Supplement: S2 File — (PDF) [file pone.0294308.s002.pdf]

Supplemental Tables

Table S1 - Spike-in Oligomers

name	RNA_Sequence
**Batch 1**	
A1	AACGCCCAGAAACGCCCCGACAAGGCGG
A2	AAAUAACCACGAAGAGCCCAAGAGAUUA
A3	GUUCGACACAGGGGAAAGGGGGGAGAGG
A4	CAUGACACACAAGGGGACAAAAGGUCGG
B1	CAUACAAAAAGAGGGAAGCAAGGGUAGA
B2	UACCGCAAGACAGGAACCGAGAGAGUUA
B3	GUUUCCCCAGAAGAGACCCGCCGCGACC
B4	GUCAGACAAGAGGGCAGAAGAGAGAUAG
**Batch 2**	
C1	CAGGGAAAACACGAAGGAGCAGAGGUUU
C2	UAAAGCAAACAAGAACAAGGCCGAUGGG
C3	UUGACGAAACCGAGACCCCCCCAGUAAC
C4	UGUUAGACGCAAGGAAAACACGAGACAC
D1	CAAGACAAGAACAGCCGGGACAAAAUAG
D2	AAACAGACGACCGCAGGACCCAAGCGUG
D3	ACAAAGAGACCCCGCAGAGCCCACAGAG
D4	CCUUACAGAGAGGGGAAGGGAACGUUCC

Table S2 - Mapping Statistics

sample	seq_depth	unique_reads	spikein_reads	rRNA_reads
C193T	50382005	2016717	31190	40308561
C193U	85058780	3193118	49737	69150869
C204T	57025679	3010568	18164	46414163
C204U	78143644	3544299	21620	63399018
C505T	48459128	2199813	7519	39707227
C505U	98746922	4167985	14694	80827933
E193T	51904657	744639	4935	44043792
E193U	136927240	1799231	12670	116181233
E204T	90395514	1248240	98469	77373538
E204U	87479545	1141768	90511	74942324
E505T	41650935	649571	17792	35208261
E505U	47206608	702012	18485	40547952
**Total**	**873380657**	**24417961**	**385786**	**728104871**
**average**	**72781721**	**2034830**	**32149**	**60675406**

Table S3 - Mapped Proportions

sample	rts_percent	kept_percent	percent_mapped	proportion_uniq
C193-MT	83.31%	16.69%	56.62%	57.36%
C193-MU	84.14%	15.86%	56.82%	56.42%
C204-MT	83.65%	16.35%	66.21%	60.92%
C204-MU	84.60%	15.40%	64.43%	59.06%
C505-MT	84.37%	15.63%	61.69%	59.79%
C505-MU	84.39%	15.61%	60.15%	57.89%
E193-MT	87.48%	12.52%	48.35%	42.04%
E193-MU	87.44%	12.56%	46.34%	42.75%
E204-MT	87.41%	12.59%	52.84%	37.87%
E204-MU	87.34%	12.66%	53.51%	36.52%
E505-MT	86.90%	13.10%	47.24%	43.64%
E505-MU	88.06%	11.94%	50.30%	41.40%

* rts_percent: percentage of reads mapped to either rRNA, tRNA, or snoRNA. kept_percent: percentage of reads kept for mapping to the genome (i.e. 1-rts_percent). percent_mapped: percentage of kept reads aligned to the genome. proportion_uniq: percentage of mapped reads that were mapped uniquely.
